# Discovery or Extinction of New *Scleroderma* Species in Amazonia?

**DOI:** 10.1371/journal.pone.0167879

**Published:** 2016-12-21

**Authors:** Iuri G. Baseia, Bianca D. B. Silva, Noemia K. Ishikawa, João V. C. Soares, Isadora F. França, Shuji Ushijima, Nitaro Maekawa, María P. Martín

**Affiliations:** 1 Departamento de Botânica e Zoologia, Universidade Federal do Rio Grande do Norte, Natal, Rio Grande do Norte, Brazil; 2 Departamento de Botânica, Instituto de Biologia, Universidade Federal da Bahia, Ondina, Salvador, Bahia, Brazil; 3 Coordenação de Biodiversidade, Instituto Nacional de Pesquisas da Amazônia, Manaus, Amazonas, Brazil; 4 Divisão de Suporte às Estações e Reservas, Instituto Nacional de Pesquisas da Amazônia, Manaus, Amazonas, Brazil; 5 Faculdade de Ciências Biológicas, Universidade Federal do Pará, Campus Universitário de Altamira, Altamira, Pará, Brazil; 6 Fungus/Mushroom Resource and Research Center, Faculty of Agriculture, Tottori University, Tottori, Japan; 7 Departamento de Micología, Real Jardín Botánico, RJB-CSIC, Madrid, Spain; University of Wisconsin Madison, UNITED STATES

## Abstract

The Amazon Forest is a hotspot of biodiversity harboring an unknown number of undescribed taxa. Inventory studies are urgent, mainly in the areas most endangered by human activities such as extensive dam construction, where species could be in risk of extinction before being described and named. In 2015, intensive studies performed in a few locations in the Brazilian Amazon rainforest revealed three new species of the genus *Scleroderma*: *S*. *anomalosporum*, *S*. *camassuense* and *S*. *duckei*. The two first species were located in one of the many areas flooded by construction of hydroelectric dams throughout the Amazon; and the third in the Reserva Florestal Adolpho Ducke, a protected reverse by the INPA. The species were identified through morphology and molecular analyses of barcoding sequences (Internal Transcribed Spacer nrDNA). *Scleroderma anomalosporum* is characterized mainly by the smooth spores under LM in mature basidiomata (under SEM with small, unevenly distributed granules, a characteristic not observed in other species of the genus), the large size of the basidiomata, up to 120 mm diameter, and the stelliform dehiscence; *S*. *camassuense* mainly by the irregular to stellate dehiscence, the subreticulated spores and the bright sulfur-yellow colour, and *Scleroderma duckei* mainly by the verrucose exoperidium, stelliform dehiscence, and verrucose spores. Description, illustration and affinities with other species of the genus are provided.

## Introduction

Amazonia is the largest and most diverse of the world’s tropical rainforests, encompassing more than 6 million km2 in nine countries of South America. According to the Rainforest Conservation Fund [1], in the rainforest most of the organisms are undescribed and unknown. Recent studies indicate at least 427 amphibians, 1294 birds, 3,000 fishes, 378 reptiles, 427 mammals, and 40,000 plant species in Amazonian rainforest [2]. Studies on fungi from the Brazilian Amazon forest have reported about 1000 species of macrofungi [3]. Knowledge of fungal diversity is amplified through advances in laboratory methodologies and computational analysis [4,5]. Molecular studies combined with morphological knowledge has led to a better delimitation of taxonomic groups, determining which morphological characters are informative, or not, so as to detect cryptic species. On the other hand, there seems to be consensus that these rainforests are reservoirs of the greatest amount of biodiversity as yet uncatalogued by science [6,7,8], which makes the destruction of the tropical rainforests the main challenge facing the discovery of fungi that are still unknown.

To ensure energy independence and exploit mineral resources, the governments of Amazonian countries are embarking on a major dam building drive on the basin’s rivers, with 191 dams finished and a further 246 planned or under construction. This rush to reap the basin’s renewable energy has come without considering the negative environmental consequences to the most speciose freshwater and terrestrial biomes of the world [9].

Brazil has emerged as one of the few countries where deforestation is falling, due to programs aimed at protecting forest areas such as blacklisting on deforestation. Critical districts with high annual forest loss are included in blacklists published regularly by the Brazilian Ministry of the Environment, and farms in those blacklists face new administrative rules to obtain licenses for clearing forests. This practice contributed to reducing the average deforestation in the years 2002 to 2012 [10,11]. Extensive projects on biodiversity studies were implemented and helped to demarcate, justify and maintain biological reserves across the country, for example, the Research Program on Biodiversity (PPBio) and the Integrated Studies Center of the Amazonian Biodiversity (CENBAM). However, the construction of increasing numbers of hydroelectric dams throughout the Amazon has led to destruction and irreversible ecological imbalance in many areas [12,13,14].

The diversity of macrofungi species present in the tropical rainforest is still insufficiently known, and Hawksworth [7] considers this biome the largest reserve of biodiversity on the planet. So far, only around 1000 species of macrofungi have been described for the Amazon forests [3]. For gasteroid fungi, 20 species have been described distributed in the states of Amazônia, Pará and Rondônia [15,16,17,18,19,20,21].

The genus *Scleroderma* was described in 1801 by Persoon and is currently included in the order Boletales [22]. In accordance with Guzmán et al. [23], *Scleroderma* is divided into three sections based on the surface structure of the basidiospores and on the presence or absence of a clamp connection: (1) *Reticulatae*, characterized by reticulated spores, (2) *Scleroderma*, with echinulate spores, and (3) *Sclerangium*, presenting subreticulated spores. Molecular studies, based on comparison of Internal Transcribed Spacer (ITS) nrDNA, confirm this classification [24,25].

This genus is distributed in tropical, subtropical and temperate regions, forming ectomycorrhizas [26]. In Brazil, there is a register of 15 species [17,27,28,29,30,31,32,33,34,35,36,37,38,39]. All of these registers were observed in exotic vegetation (*Pinus* spp, *Eucalyptus* spp, etc.), with the exception of *S*. *minutisporum* Baseia, Alfredo & Cortez and *S*. *dunensis* BDB Silva, Sulzbacher, Grebenc, Baseia & MP Martín, which were found in native vegetation of the Amazon rainforest [17,39]. *Scleroderma tenerum* Berk & M.A. Curtis, and *S*. *tuberoideum* Speg. are considered synonymous with *S*. *nitidum* Berk. and *S*. *albidum* Pat. & Trab., respectively [23,30].

On March 28, 2015, some of the authors of this article (NKI, IFF, SU and NM) visited Camassú, one of about 50 islands that would be flooded due to construction of Belo Monte Dam; they collected a number of *Scleroderma* specimens that were not possible to assign to any known species.

This work describes novelties of the genus *Scleroderma* from the Amazon rainforest with analyses based on morphological and molecular data.

## Material and Methods

### Collections studied

The species were collected from native vegetation of the Brazilian Amazon rainforest, and were deposited in Brazilian and Spanish collections: UFRN (Rio Grande do Norte), INPA (Amazonas) and MA-Fungi (Madrid). Data of collections studied are included in Table 1. All necessary permits were obtained for studies issued by the Curators of the Herbaria (reference document number UFRN-02/2015, INPA-13/2015).

**Table 1:** *Scleroderma* species included in the molecular analyses with their herbarium and/or isolate numbers, country and GenBank accession numbers of internal transcribed spacer (ITS) nuclear ribosomal DNA. (n.d.: no data). In bold, new species described in this study.

Species	Herbarium voucher; isolate	Country	GenBank Acc. N° ITS
***S*. *anomalosporum***	**UFRN-Fungos 2790**	**Brazil**	**KX792084**
*S*. *areolatum*	OSC36088; JMP0054	USA	EU819518
*S*. *areolatum*	OSC38819; JMP0080	USA	EU819438
*S*. *areolatum*	OSC122632	USA	FM213351
*S*. *areolatum*	PDD75096	USA	FM213352
*S*. *areolatum*	E00278286	USA	FM213353
*S*. *areolatum*	MEL1054289	USA	GQ166910
*S*. *areolatum*	02MCF4202	Macedonia	HF933231
*S*. *bermudense*	BZ3961	Belize	EU718118
*S*. *bovista*	n.d	Japan	AB099901
*S*. *bovista*	n.d	Japan	AB211267
*S*. *bovista*	K (M) 105588	USA	EU784409
S. bovista	RT00034	USA	EU819517
*S*. *bovista*	BCN-MPM1989	Spain	FM213340
*S*. *bovista*	MJ6006	Hungary	FM213341
*S*. *bovista*	K80S09	New Zealand	GQ267487
*S*. *bovista*	MCF 01/168; 01MCF168Sbov	Macedonia	HF933234
*S*. *bovista*	MCF 05/788; 05MCF788Sbov	Macedonia	HF933235
*S*. *bovista*	MCF 05/5304; 05MCF5304Sbov	Macedonia	HF933236
*S*. *bovista*	MCF 06/5993; 06MCF5993Sbov	Macedonia	HF933240
*S*. *bovista*	MCF 09/11184; 09MCF1118	Serbia	HF933242
*S*. *bovista*	MA-Fungi 87407; MPM3241	Cape Verde	KX017590
***S*. *camassuense***	**UFRN-Fungos 2793**	**Brazil**	**KX792085**
*S*. *capeverdeanum*	MA-Fungi 87406; MPM3238	Cape Verde	KU747110
S. *cepa*	SOC541	USA	DQ453694
*S*. *cepa*	MCA242	North Carolina, USA	EU718117
*S*. *cepa*	n.d; UNSCL_7	Thailand	FM213343
*S*. *cepa*	E00278296; CEPSCL_5	USA	FM213355
*S*. *citrinum*	K (M) 17485	UK	EU784413
*S*. *citrinum*	K (M) 53906	UK	EU784414
*S*. *citrinum*	(root tip)	USA	FJ824090
*S*. *citrinum*	SCL3; UNSCL_2	UK	FM213333
*S*. *citrinum*	SCL5; UNSCL_3	UK	FM213334
*S*. *citrinum*	SCL7; UNSCL_4	UK	FM213335
*S*. *citrinum*	E00278300; CITSCL_1	USA	FM213344
*S*. *citrinum*	n.d; CITSCL_2	USA	FM213345
*S*. *citrinum*	F-PRL5772	USA	GQ166907
*S*. *dictyosporum*	IR215	Burkina Faso	FJ840443
*S*. *dictyosporum*	SD-4901	Burkina Faso	FJ840449
***S*. *duckei***	**UFRN-Fungos 2794**	**Brazil**	**KX792086**
***S*. *duckei***	**UFRN-FUngos 2795**	**Brazil**	**KX792087**
*S*. *dunensis*	UFRN-Fungos 2033	Brazil	KU747112
*S*. *dunensis*	UFRN-Fungos 1359	Brazil	KU747113
*S*. *dunensis*	UFRN-Fungos 1661	Brazil	KU747114
*S*. *dunensis*	UFRN-Fungos 2549	Brazil	KU747115
*S*. *dunensis*	UFRN-Fungos 2551	Brazil	KU747116
*S*. *dunensis*	UFRN-Fungos 2035	Brazil	KU747117
*S*. *dunensis*	UFRN-Fungos 2553	Brazil	KU747118
*S*. *dunensis*	UFRN-Fungos 2501	Brazil	KU747119
*S*. *dunensis*	UFRN-Fungos 2499	Brazil	KU747120
*S*. *dunensis*	UFRN-Fungos 2206	Brazil	KU747121
*S*. *meridionale*	CCMA; M. Soussi 21	Spain	AY935514
*S*. *meridionale*	MCF 05/5505; 05MCF5505Smer	Macedonia	HF933238
*S*. *meridionale*	MCF 05/5505; 05MCF5505Smer	Macedonia	HF933239
*S*. *michiganense*	JMP0081	USA	EU819439
*S*. *michiganense*	JMP0083	USA	EU819441
*S*. *michiganense*	E00278306	USA	FM213346
*S*. *michiganense*	E00278311	USA	FM213347
*S*. *michiganense*	E00278309	USA	FM213348
*S*. *nastii*	NAST-FB11	Nepal	KJ740390
*S*. *nitidum*	UFRN-Fungos 2034	Brazil	KU759904
*S*. *nitidum*	UFRN-Fungos 2550	Brazil	KU759906
*S*. *nitidum*	UFRN-Fungos 1759	Brazil	KU759907
*S*. *nitidum*	UFRN-Fungos 2219	Brazil	KU759908
*S*. *nitidum*	UFRN-Fungos 2500	Brazil	KU759909
*S*. *patagonicum*	CORD; Trappe 26236	Argentina	HQ688788
*S*. *patagonicum*	CORD; Trappe 26232	Argentina	HQ688789
*S*. *polyrhizum*	POLSCL1	USA	FM213349
*S*. *polyrhizum*	POLSCL2	USA	FM213350
*S*. *septentrionale*	AWW218	Massachussetts, USA	EU718121
*S*. *septentrionale*	SEPSCL_1	USA	FM213337
*S*. *septentrionale*	SEPSCL3_C	USA	FM213338
*S*. *septentrionale*	BOVSCL_2	USA	FM213339
*S*. *septentrionale*	UNSCL_5	USA	FM213342
*S*. *sinnamariense*	SCLK4; SINSCL_1	Thailand	FM213356
*S*. *sinnamariense*	SCLP3; SINSCL_2	Thailand	FM213357
*S*. *sinnamariense*	SCLN; SINSCL_3	Thailand	FM213358
*S*. *sinnamariense*	SCLY5; SINSCL_4	Thailand	FM213359
*S*. *sinnamariense*	SC1; SINSCL_5	Thailand	FM213360
*S*. *sinnamariense*	SCLD1; SINSCL_6	Thailand	FM213361
*S*. *sinnamariense*	SINSCL_7; SINSCL_7	Thailand	FM213362
*S*. *sinnamariense*	SINSCL_8; SINSCL_8	Thailand	FM213363
*S*. *sinnamariense*	SINSCL_9; SINSCL_9	Thailand	FM213364
*S*. *sinnamariense*	NAST-FB11	Thailand	HQ687222
*S*. *suthepense*	strain CMU55-SC2	Thailand	JX205215
*S*. *verrucosum*	BCN-MPM2605	Spain	AJ629886
*S*. *verrucosum*	K (M) 54373	England	EU784412
*S*. *verrucosum*	K (M) 30670	England	EU784415
*S*. *verrucosum*	BCN-MPM 2525; CEPSCL_2	Spain	FM213354
*S*. *verrucosum*	MCF 07/7984; 07MCF7984Sver	Macedonia	HF933232
*S*. *verrucosum*	MCF 08/10124; 08MCF10124Sver	Macedonia	HF933233
*S*. *verrucosum*	MCF 89/4709; 89MCF4709Scitcf	Macedonia	HF933237
*S*. *verrucosum*	MCF 06/7265; 06MCF7265Sver	Macedonia	HF933241
*S*. *xanthochroum*	AWW254	Malaysia	EU718126
*S*. *yunnanense*	KUN-HKS79633A; isolate Ji001A	China	JQ639040
*S*. *yunnanense*	KUN-HKS79633B; isolate Ji001B	China	JQ639041
*S*. *yunnanense*	KUN-HKS79633C; isolate Ji001C	China	JQ639042
*S*. *yunnanense*	KUN-HKS79633D; isolate Ji001C	China	JQ639043
*S*. *yunnanense*	KUN-HKS79664B; isolate Ji002B	China	JQ639045
*S*. *yunnanense*	KUN-HKS79665; isolate Ji003	China	JQ639046
*Scleroderma* sp.1 (*S*. *polyrhizum*)	Strain 11–3	China	HM237173
*Scleroderma* sp.1 (*S*. *aurantiacum*)	Strain 8–5	China	HM237174
*Scleroderma* sp. 2 *(S*. *septentrionale)*	J. Nitare 12.9.1986; SEPSCL2	Sweden	FM213336
*Scleroderma* sp. 3 (*S*. *verrucosum*)	SV-5602	Burkina Faso	FJ840461
*Scleroderma* sp. 4	UFRN 2792	Brazil	KX792088
*Pisolithus arhizus*, outgroup	BCN-MPM 2676	Spain	FM213365

### Morphological analysis

The morphological analyzes with dry material followed preliminary studies [23,30,40,41,42,43], and were performed in the fungal biology laboratory at the Universidade Federal do Rio Grande do Norte. Measurements were performed using a ruler attached to the microscope with smallest divisions at 1 mm. For microscopic analysis hand-cut sections of the layers of the peridium and spores, mounted in 5% KOH, Melzer's reagent and Congo Red were examined with the light microscope. The standardization of the colors followed Kornerup and Wanscher [44].

### Molecular analyses

Samples for DNA extraction were excised from dry basidiomes. To avoid contamination by other fungi, pseudotissues were taken from the inner part of the basidiome. DNA extraction, amplification, and sequencing of the ITS regions including the 5.8S of the ribosomal RNA gene cluster followed the protocols mentioned by Phosri et al. [24]. The ITS regions were amplified with Ready-To-Go^TM^ PCR Beads (GE healthcare Life Sciences, NJ, USA), using the primers ITS1F [45] and ITS4 [46], and the cycling protocol described in Martín and Winka [47]. Aliquots of the purified products were mixed separately with the direct and reverse primers before sending them to Macrogen (South Korea) for sequencing. Consensus sequences were assembled using Sequencher software (Gene Codes Corporation Inc, Ann Arbor, Michigan, USA). Previous to the alignment, sequences were compared with homologous sequences from the EMBL/GenBank/DDBJ [48] using the BLASTn algorithm [49]. All new sequences have been deposited on the EMBL-EBI database and their accession numbers are presented in Table 1.

Using SEQAPP software (PerkinElmer Applied Biosystems), multiple sequence alignments were performed of the consensus sequences obtained in this study and homologous sequences from the EMBL/GenBank/DDBJ, (http://www.ncbi.nlm.nih.gov/entrez/) (Phosri et al. [24], Rusevska et al. [25], and Crous et al. [39]) shown in Table 1. The alignment was optimized visually. Alignment gaps were indicated as “-” and ambiguous nucleotides were marked as “N”.

The alignment was analyzed using the programms PAUP 4.0a147 [50], MrBAYES 3.2.2 [51] and RAxML [52] using the CIPRES portal (http://www.phylo.org/portal2/) [53]. *Pisolithus arhizus* FM213365 was used as outgroup, since this species is closely related to *Scleroderma* [54]. First, a parsimony analysis under a heuristic search was conducted. Gaps were treated as missing data. The tree branch robustness was estimated by bootstrap (MP-BS) analysis [55] employing 10000 replicates, using the fast-step option. The starting branch lengths were obtained using the Roger-Swofford approximation method and the starting trees for branch swapping were obtained by stepwise addition. The tree bisection-reconnection (TBR) branch-swapping algorithm was used with the Multitrees options. The data were further analyzed using a Bayesian approach [56,57]. The posterior probabilities (PP) were approximated by sampling trees using the MCMC method. The Bayesian analysis was performed assuming the general time reversible model [58] including estimation of invariant sites and assuming a discrete gamma distribution with six rate categories (GTR+I+G). A run with 2M generation starting with a random tree and employing 12 simultaneous chains was executed. Every 100th tree was saved into a file. The log-likelihood scores of sample points were plotted against the number of generations using TRACER 1.0 (http://evolve.zoo.ox.ac.uk/software.html) to determine that stationarity was achieved when the log-likelihood values of the sample points reached a stable equilibrium value [51]. The initial 1000 trees were discarded as a burn-in before calculating posterior probabilities (PP). Using the “sumt” command of MrBAYES, the majority-rule consensus tree was calculated from 19K trees sampled after reaching likelihood convergence to calculate the posterior probabilities. A third maximum likelihood bootstrapping analysis was performed with RAxML 7.2.8 [52], using the default parameters as implemented on the CIPRES NSF XSEDE resource with bootstrap statistics calculated from 1000 bootstrap replicates (ML-BS) under GTR + I + G model of evolution.

The phylogenetic tree was drawn with the program TreeView [59] and edited in Adobe Illustrator CS3; names of clades and subclades are according to Phosri et al. [24], Rusevska et al. [25], and Crous et al. [39]. A combination of MP-BS, ML-BS, and PP was used to assess confidence for a specific node [60,61].

### Nomenclature

The electronic version of this article in Portable Document Format (PDF) in a work with an ISSN or ISBN will represent a published work according to the International Code of Nomenclature for algae, fungi, and plants, and hence the new names contained in the electronic publication of a PLOS ONE article are effectively published under that Code from the electronic edition alone, so there is no longer any need to provide printed copies.

In addition, new names contained in this work have been submitted to MycoBank, from where they will be made available to the Global Names Index. The unique MycoBank number can be resolved and the associated information viewed through any standard web browser by appending the MycoBank number contained in this publication to the prefix at http://www.mycobank.org/MB. The online version of this work is archived and available from the following digital repositories: PubMed Central, LOCKSS and Digital-CSIC.

## Results

### Molecular studies

**Fig 1:**
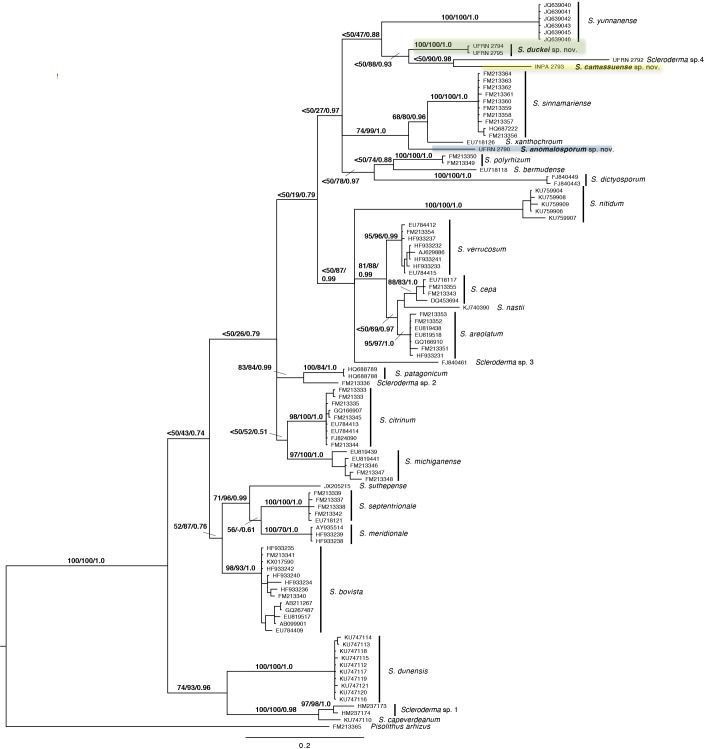
Phylogenetic tree obtained from Bayesian analysis of *Scleroderma* species. Numbers above branches are parsimony bootstrap (MP-BS), maximum likelihood bootstrap (ML-BS) and posterior probability (PP) values. The position of the three new species described in this paper are marked in colours, indicated by the herbarium number (UFRN = UFRN-Fungos); the rest of branches indicated with their respective GenBank accession numbers, indicated in Table 1.

The sequences obtained from Amazonian specimens have been deposited within EMBL (http://www.ebi.ac.uk/embl) with the accession numbers indicated in Table 1. The topologies of the three analyses performed (Maximum parsimony, Maximum likelihood and Bayesian) were similar to each other; the 50% majority-rule consensus tree from the Bayesian analysis is shown in Fig 1 with MP-BS, ML-BS and PP on branches. At least 20 clades can be assigned to *Scleroderma* species already defined in Crous et al. [39]. As indicated in Fig 1, the Amazonian specimens grouped into three different clades.

The sequence from collection UFRN-Fungos 2790 is the sister group of the clade formed by *Scleroderma sinnamariense* Mont. and *S*. *xanthochroum* Watling & K.P. Sims. This relationship is very well supported (MP-BS = 74%, ML-BS = 99%, PP = 1.0); although, the specimens of collection UFRN-Fungos 2790 show unusual spore ornamentation for a *Scleroderma*: small granules under SEM; whereas in *S*. *sinnamariense* spores are echinulate and in *S*. *xanthochroum* reticulated [62].

The rest of the specimens grouped together with low MP-BS support (< 50%), although distributed in two different clades. One clade contained the two collections UFRN-Fungos 2794 and UFRN-Fungos 2795 with significant support (MP-BS = 100%, ML-BS = 100%, PP = 1.0); these collections are from the Reserva Florestal Adolfo Duke, and the specimens show spores slightly spiny under LM. In the other clade, the collections INPA 271114 and UFRN-Fungos 2792, grouped together, with low MP-BS support (< 50%), and strong ML-BS (90%) and PP (0.98); the number of differences between the sequences of these collections (Fig 1) suggests to us that they could belong to two different taxa, but specimen UFRN-Fungos 2792 was not in good enough condition to perform a complete morphological analysis.

Based on morphological and molecular analyses, *S*. *anomalosporum* (UFRN-Fungos 2790), *S*. *camassuense* (UFRN-Fungos 2793), and *S*. *duckei* (UFRN-Fungos 2794 and UFRN-Fungos 2795) are proposed as new species.

### Taxonomy

***Scleroderma anomalosporum*** Baseia, B.D.B. Silva & M.P. Martín sp. nov., Mycobank MB 818095

Etymology. In reference to unusual spores compared to the pattern of spores of the genus *Scleroderma*.

Holotype. Brasil, Pará, Altamira, Ilha Camassú, S03°16'46.0" W052°12'17.1", 28 Mar. 2015, leg. N.K. Ishikawa & I.F. França (UFRN-Fungos 2790; ITS nrDNA sequence Acc. Number KX792084).

Isotypes. INPA 271001; MA-Fungi 89305

Diagnosis. Basidiomata epigeous, sessile, opening by stellate dehiscence, up to 115 mm diam, surface reticulated. Peridium 450–600 mm thick, consisting of three layers. Gleba when mature protected by the inner layer of peridium. Basidiospores 3.5–5.3 × 3.8–5.4 μm diam, globose to subglobose, smooth under LM, with small granules on the surface under SEM.

**Fig 2:**
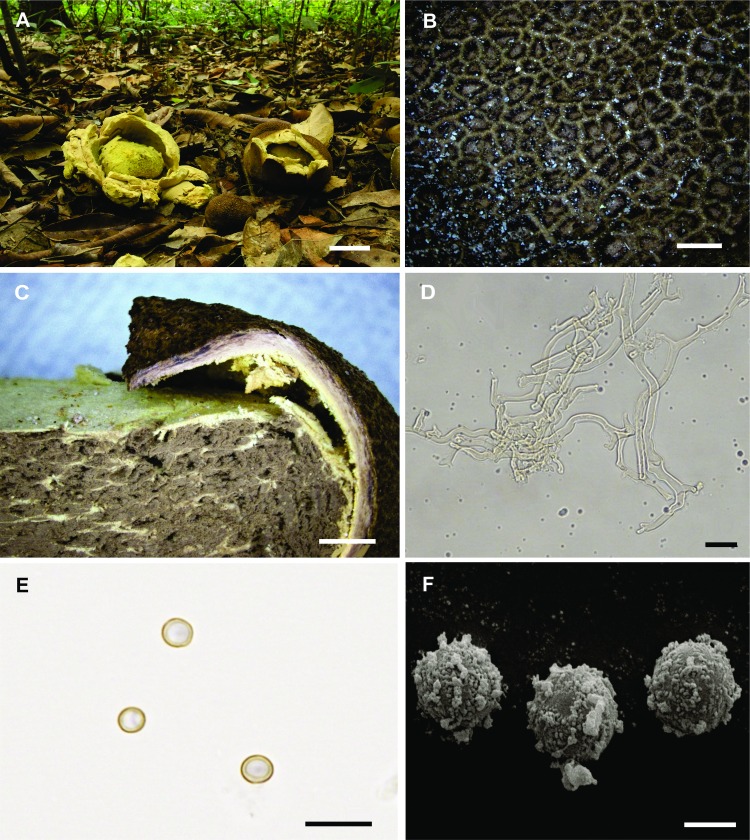
*Scleroderma anomalosporum*. (A) Fresh basidiomata in the field, bar = 30 mm. (B) Detail of reticulation in exoperidium of young basidioma, bar = 2 mm. (C) Basidioma cut away side view, bar = 2 mm. (D) Exoperidium hyphae, bar = 20 μm. (E) Basidiospores under LM, bar = 10 μm. (F) Basidiospores under SEM, bar = 2 μm.

Description. Basidiomata epigeous, sessile, subglobose when closed, up to 90 mm diam × 45 mm high; when mature, stellate dehiscence forming 5–7 irregular branches, the expanded basidiomata up to 115 mm diam × 75 mm high, with rhizomorphs aggregated at the base (Fig 2A). Surface reticulated, brown (5F6, 5F7) to dark brown (6F6) at maturity, with aggregated soil particles (Fig 2B). Peridium 450–600 mm thick, with three layers (Fig 2C): the outer layer made of cylindrical hyphae, yellowish in KOH, 2.5–6.5 μm diam, walls up to 1.0 μm thick, winding (Fig 2D); the middle layer consists of cylindrical hyphae, with rounded ends at the surface, hyaline in KOH, 4.5–16 μm diam, walls up to 2.5 μm thick; and inner layer pale yellow (3A3), composed of interwoven cylindrical hyphae, hyaline in KOH, 4.0–6.5 μm diam, walls up to 1.0 μm thick, clamp connections rare. Gleba when mature greyish brown (6E3), compact to powdery at maturity, protected by the inner layer of peridium. Basidiospores 3.5–5.3 × 3.8–5.4 μm diam including ornamentation, globose to subglobose, hyaline to yellowish in KOH, smooth under LM (Fig 2E), with small granules on the surface under SEM (Fig 2F).

Remarks. *Scleroderma anomalosporum* is characterized mainly by the smooth spores under LM in mature basidiomata and the large size of the basidiomata, being capable of achieving up to 120 mm in diameter when expanded, and the stelliform dehiscence. In accordance with Guzmán et al. [22], smooth spores in the genus *Scleroderma* are found in immature basidiomes, and when mature, the spores vary between reticulated, subreticulated and echinulate. The spores of *S*. *anomalosporum* under SEM present small, unevenly distributed granules, a characteristic not observed in other species of the genus. *Scleroderma polyrhizum* (J.F. Gmel) Pers. and *S*. *texense* Berk. present basidiomata that can reach up to 150 mm and 140 mm, respectively, when expanded. However, they have larger spores (7–11 μm in diameter) than *S*. *anomalosporum*, and different ornamentation: subreticulated and lightly echinulate spores in *S*. *polyrhizum* [23,30], and reticulated in *S*. *texense* [23,30].

***Scleroderma camassuense*** M.P. Martín, Baseia & B.D.B. Silva sp. nov., Mycobank MB 818096

Etymology. In reference to the type locality in the state of the Pará.

Holotype. Brasil, Pará, Altamira, Ilha Camassú, S03°16'46.0" W52°12'17.1", 28 Mar. 2015, leg. N.K. Ishikawa & I.F. França (UFRN-Fungos 2793, ITS nrDNA sequence Acc. Number KX792085).

Isotype. INPA 271114

Diagnosis. Basidiomata epigeous, sessile or pseudostipitate, opening by a dehiscence irregular to stellate, up to 20 mm diam, surface scaly to verruculose. Peridium up to 0.5 mm thick, consisting of three layers. Basidiospores 6.4–8.0 × 5.6–7.5 μm diam, globose to subglobose, subreticulated under LM, irregular reticulum under SEM.

**Fig 3:**
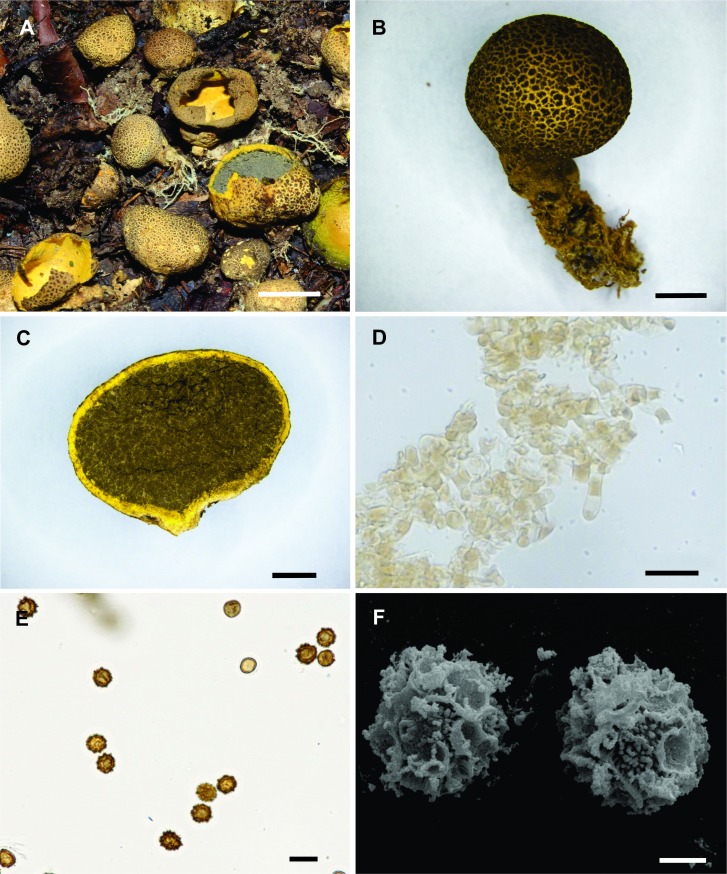
*Scleroderma camassuense*. (A) Fresh basidiomata in the field, bar = 10 mm. (B) Detail of verrucose exoperidium surface, bar = 2 mm. (C) Basidioma cut away side view, bar = 2 mm. (D) Exoperidium hyphae, bar = 20 μm. (E) Basidiospores under LM, bar = 10 μm. (F) Basidiospores under SEM, bar = 2 μm.

Description. Basidiomata epigeous, sessile or pseudostipitate, globose to subglobose when closed, up to 14 mm diam × 12 mm high; when mature, irregular to stellate dehiscence forming 4–6 irregular branches, expanded up to 20 mm diam × 11 mm high (Fig 3A). Generally, there are yellow (2A6) rhizomorphs or mycelium attached at the base (Fig 3B). Surface scaly to verruculose, dark brown (6F4) at maturity, with soil particles aggregated (Fig 3B). Peridium 0.5 mm thick, consisting of three layers (Fig 3C); the outer layer made of oleoacanthohyphae with yellowish contents in KOH, 5.5–10.5 μm diam, walls up to 1.0 mm thick (Fig 3D); the middle layer sulphur yellow (1A5), composed of interwoven cylindrical hyphae, hyaline in KOH, 3.5–6 μm diam, walls up to 1 μm thick; and the inner layer sulphur yellow (1A5), composed of pseudoparenchymatous cells, hyaline in KOH, 26–57 × 13–35.5 μm, walls up to 1 mm thick. Gleba when mature brown (5E4) to greyish brown (6F3), compact to powdery at maturity. Basidiospores 6.4–8.0 × 5.6–7.5 μm diam including ornamentation, globose to subglobose, brownish in KOH, subreticulated under LM (Fig 3E), irregular reticulum under SEM (Fig 3F).

Remarks. *Scleroderma camassuense* is characterized mainly by the irregular to stellate dehiscence, the subreticulated spores and the yellow sulfur colour. *Scleroderma sinnamariense*, *S*. *verrucosum* (Bull.) Pers., *S*. *citrinum* Pers. and *S*. *uruguayense* (Guzmán) Guzmán also present a dark yellow peridium, but can be distinguished by the size of the larger basidiomata (up to 45 mm in diameter) and the presence of pilocystidia in the external layer of the peridium in *S*. *sinnamariense* [30,42]; by the larger and echinulate spores (9–12 μm in diameter) in *S*. *verrucosum* [43]; and by the larger and reticulated spores (11–14 μm in diameter) in *S*. *citrinum* and *S*. *uruguayense* [30,43].

Subreticulated spores and stellate dehiscence are also observed in *Scleroderma bermudense* Coke*r*, *S*. *floridanum* Guzmán, *S*. *stellatum* Berk., *S*. *polyrhizum* and *S*. *texense*. However, they can be differentiated from one another by the larger basidiomata (up to 34 mm in diameter), whitish or light brown peridium, and presence of interlaced fibrils in *S*. *bermudense* [43]; by the larger spores (8.8)10.4–13.6(–16) μm and flaky surface of the exoperidium in *S*. *floridanum* [29]; by the echinulate exoperidium in *S*. *stellatum* [43]; by the larger spores (6)7.2–9.6(–12) μm and larger basidiomata in *S*. *polyrhizum* and *S*. *texense* with 140 mm and 150 mm, respectively [23,30].

***Scleroderma duckei*** B.D.B. Silva, M.P. Martín & Baseia sp. nov., Mycobank MB 818097

Etymology. In reference to the type locality, Reserva Florestal Adolpho Ducke

Holotype. Brasil, Amazonas, Manaus, Reserva Adolpho Ducke, S02°57'37.3" W59°55'55.1", 21 Mar. 2015, leg. N.K. Ishikawa & J.V.C. Soares (UFRN-Fungos 2794, ITS nrDNA sequence KX792086).

Isotype. INPA 272127.

Diagnosis. Basidiomata epigeous, sessile, opening by a small stellate dehiscence, up to 25 mm diam, surface verrucose. Peridium up to 0.5 mm thick, consisting of three layers. Basidiospores 5.7–7.1 × 5.7–7.0 μm diam, globose to subglobose, slightly spiny under LM, regularly grouped warts under SEM.

**Fig 4:**
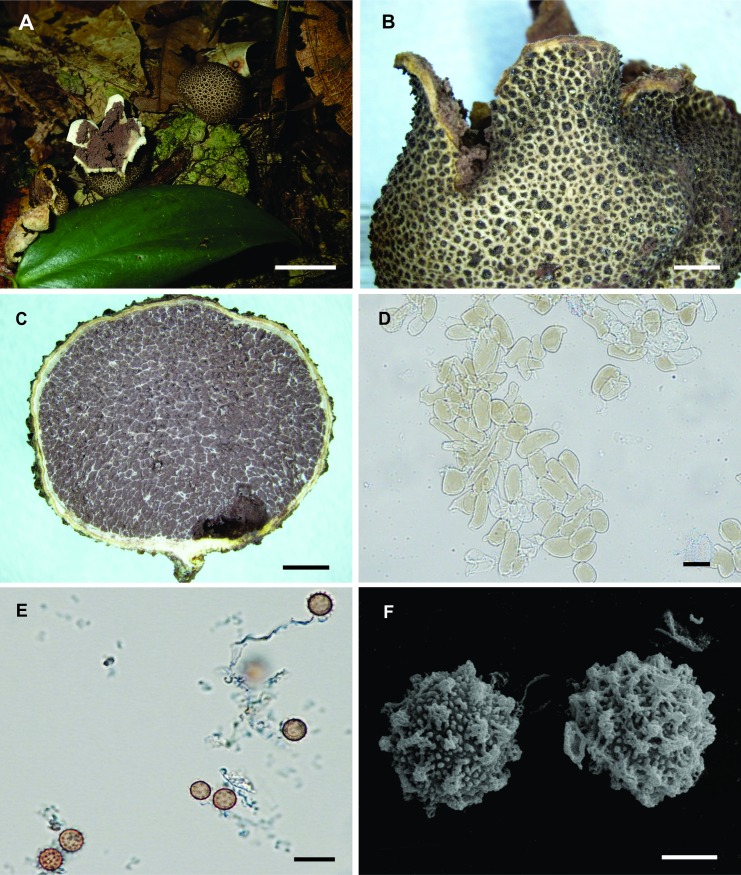
*Scleroderma duckei*. (A) Fresh basidiomata in the field, bar = 20 mm. (B) Detail of verrucose exoperidium surface, bar = 2 mm. (C) Basidioma cut away side view, bar = 2 mm. (D) Exoperidium hyphae, bar = 20 μm. (E) Basidiospores under LM, bar = 10 μm. (F) Basidiospores under SEM, bar = 2 μm.

Description. Basidiomata epigeous, sessile, when closed globose to subglobose, up to 20 mm diam × 8 mm high; when mature, small stellate dehiscence that form 5–6 irregular branches, expanded up to 25 mm diam x 16 mm high, with rhizomorphs aggregated at the base (Fig 4A). Surface verrucose, dark brown (6F6, 6F4) at maturity, with aggregated soil particles (Fig 4B). Peridium up to 0.5 mm thick, consisting of three layers (Fig 4C): the outer layer is composed of pseudoparenchymatous irregular cells, yellowish in KOH, 13.5–47.5 × 13–22 μm, walls up to 2.0 μm thick (Fig 4D); the middle layer consists of cylindrical hyphae, clamp connections rare, hyaline in KOH, 5–20 μm diam, walls up to 1.9 μm thick; and the inner layer composed of interwoven cylindrical hyphae, hyaline in KOH, 5–15 μm diam, walls less than 1 μm thick, with clamp connections. Gleba when mature dark brown (7F4), compact to powdery at maturity. Basidiospores 5.7–7.1 × 5.7–7.0 μm diam including ornamentation, globose to subglobose, brownish in KOH, slightly spiny under LM (Fig 4E), regularly grouped warts under SEM (Fig 4F).

Other material studied. Brasil, Amazonas, Manaus, Reserva Adolpho Ducke S02°57'37.3" W59°55'55.1", 21 Mar. 2015, N.K Ishikawa & J.V.C. Soares (UFRN-Fungos 2795 ITS nrDNA Acc. Number KX792087).

Remarks. *Scleroderma duckei* is characterized mainly by the verrucose exoperidium, stelliform dehiscence, and verrucose spores, with warts regularly grouped on the surface of the wall and with small spines visible in optical microscopy. *Scleroderma bermudense*, *S*. *minutisporum*, *S*. *sinnamariense*. and *S*. *stellatum* also present small spores, 5–10 μm [42], 4–7 μm [16], 7–9 μm [42], and (5–)6-7(–9) μm [43], respectively. However, they differentiate themselves from one another by the whitish, light brown or light grey peridium and the presence of interlaced fibrils in *S*. *bermudense* [43]; by the spores with an irregular reticule and velutinous or woodruff exoperidium in *S*. *minutisporum* [17]; by the sulfur-yellow peridium and spores with well-developed reticule in *S*. *sinnamariense* [42]; and by the larger basidiomata (up to 45 mm when expanded), echinulate peridium surface and spores forming a subreticule in *S*. *stellatum* [43].

## Discussion

The centers of endemism in Amazonia are well established for animals (vertebrates) and plants [63], and the type locality of the two new *Scleroderma* species, *S*. *anomalosporum* and *S*. *camassuense*, are within these areas of high endemism. According to Haffer [64], there are many hypotheses proposed to explain barrier formation separating populations and causing the differentiation of species in Amazonia during the course of geological history based on different factors. Among them there is the river hypotheses, due to the barrier effect of Amazonian rivers. Several anthropogenic activities such as accelerated deforestation and flooded areas from the construction of dams, contribute to the rapid habitat degradation in the Central Amazon. These events, and the disorderly growth of cities in Northern Brazil, in association with climate changes, makes the scientific community recognize the urgency in learning about the biodiversity in this kind of megadiverse area, before the current species become extinct due to human activities [65,66,67,68,69].

The results obtained through the morphological and molecular studies show an interesting species richness of *Scleroderma* with peculiar morphology, as in the case of *S*. *anomalosporum* that presents unusual spores in comparison with other species of this genus. The sequences obtained from Amazonian specimens as indicated in Fig 1, show that the Amazonian specimens are grouped in three different clades and well-supported to be considered independent taxa. *Scleroderma anomalosporum* and *S*. *camassuense* are described from Camassú island (type locality) that is now under the unnatural level of the Xingu River waters; and the third new species, *S*. *duckei*, is described from a protected reserve by the INPA. After decades of collecting in rainforests, these three species have not been described before, and they could be endemic to their respective habitats. In a recent study of diversity and distribution of ectomycorrhizal fungi in white-sand forests in Amazonia (along the Cuieiras river) and French Guiana [70], only *S*. *minutisporum* [17] is mentioned. In Amazonia, species have restricted distribution [71], being very sensitive to any changes in their habitats [72,73]. Based on complete checklists of published flora data from Brazil (Ducke Reserva), French Guiana (Saül region) and Peru (the Iguits area), Hopkins [74] pointed that it is extremely rare to found a species in any locality; based on this, authors claim that the conservation of Amazonian biodiversity requires actions in all landscapes, not only in protected areas [75].

There are enormous shortfalls in biological knowledge of the Amazon rainforest [76]. Although great efforts to develop international research networks are gathering existing data about species diversity, the number of species that the Amazonia contains it is not yet known [77]. With the exiting data, how can we best protect Amazonia’s biodiversity? Authors agree that the more knowledge we have, the better prepared we will be to protect and maintain the Amazonian biodiversity [75]. However, the scientific process of describing new species is slow compared with the high rates of destructions of natural landscapes [75]. At least, two of the species here described, *Scleroderma anomalosporum* and *S*. *camassuense* could already be extinct, since their collections sites are now under water.

What do we already know about fungal extinctions and how much does it matter? When looking through public databases for studies related to fungus (or fungi) and extinction, almost all papers listed concern pathogenic fungi associated, for example, with the risk of extinction and decline of amphibians [78,79], bees [80], bats [81,82,83], or with recent hypotheses about their role in mass extinction of dinosaurs [84], also some related to invasive plants diseases [85]. However, the information related to the extinction of fungi *per se* is limited [86]. It is well known that fungi play a key role in all biomes as organic matter decomposers and, the great contribution that ectomycorrhizal fungi make to plant nutrition in infertile soils [87], such as *Scleroderma* species. Many *Scleroderma* species found in Brazil forms ectomycorrhiza with introduced *Pinus* spp. and *Eucalyptus* spp.; however, two species where located in native vegetation of the Amazon rainforest [17,39], as well as, the three species described here. The extinction of any mycorrhizal fungi can be a very important matter to the associate plant.

Our results support the designation of the Amazon Forest as a hotspot [88] with high diversity and several taxa still unknown to Science. Inventory studies are urgent, mainly in the areas most endangered by human activities, where species could be in risk of extinction before being described and named.
